# Assessment of Physicochemical and Microbiological Quality of Public Swimming Pools in Addis Ababa, Ethiopia

**DOI:** 10.2174/1874285801711010098

**Published:** 2017-06-21

**Authors:** Kokebe Yedeme, Melese Hailu Legese, Almaz Gonfa, Somson Girma

**Affiliations:** 1 St. Peter’s Specialized Hospital, Addis Ababa, Ethiopia; 2 Department of Medical Laboratory Science, School of Allied Health Sciences, College of Health Science, Addis Ababa University, Addis Ababa, Ethiopia; 3 Ethiopian Health and Nutrition Research Institute, Addis Ababa, Ethiopia

**Keywords:** Microbial quality, Physicochemical, Safety, Swimming pools, Addis Ababa, Ethiopia

## Abstract

**Background:**

From swimming pools, bathers may acquire many potential pathogens or may be affected by the physicochemical characteristics of water used during bathing. Hence, this study aimed at assessing the physicochemical and microbiological quality of public swimming pools located at different hotels and recreation center in Addis Ababa, Ethiopia.

**Method:**

A cross sectional study was carried out from February to May, 2016. Nine hotels and one recreation center which recognized to have public swimming services were included. A total of 60 swimming pool water samples from 10 swimming pools were collected at deeper, shallow and intake point twice on a weekly basis using a 250 ml sterile bottle containing sodium thiosulphate. PH, residual chlorine and temperature of samples were recorded at the time of collection. Sample containing bottles were transported in ice box to microbiological laboratory and analyzed on the same day. Standard cultural and biochemical methods were used for isolation and characterization of the main microbial groups. Total viable count, total coliform count, fecal coliform count and *E. coli* were determined. Data was analyzed using SPSS Version 20.

**Results:**

Average PH and temperature of swimming pool water samples were 7.1 and 29^o^C respectively. Of all analyzed water samples, 58.4% (n=35/60) of them had PH range of 7.2-7.8, 58.3% (n=35/60) of samples had temperature in the range of 21^o^C-32^o^C and 25% (n=15/60) of water samples had residual chlorine in the range of 2-3mg/l. 73.3% (n=44/60) of the samples had a total viable count below 200 MPN/ml and 70% (n-42/60) of the samples had Total Coliform Count values less than 2 MPN/100 ml. Moreover, 66.7% (n=40/60) of the samples had fecal coliform counts falling below 1 MPN /100 ml. *E. coli* was absent in 70% (n=42/60) of the samples while it was present in 30% (n=18/60) of the samples.

**Conclusion:**

PH, residual chlorine and temperature value of majority of the swimming pools’ water samples were within the acceptable limit. Regarding microbial quality, most swimming pools’ water samples complied to the WHO standard. Swimming pools that did not comply to the standard both in physicochemical levels and microbial quality need improvement due to their significant health implication.

## INTRODUCTION

The use of public swimming pools for recreational activities, rehabilitative treatment or sport is growing worldwide [1, 2]. Swimming service givers all around the world use different forms of chlorine as disinfectant to clean swimming pools as sodium hypochlorite, dichlor or trichlor (3).When any of these pool chemicals are used, it is very important to keep the pH of the pool in the range of 7.2 to 7.6 [3, 4]. Physicochemical factors such as pH are very influential and should be controlled to ensure efficient disinfection, to avoid damage to the pool fabric and ensure user comfort [2, 3]. For example, if the water pH is alkaline, chlorine antiseptic performance will decrease. In addition, pools which have a high temperature provide the grounds for fungal contamination [2].

Recreational waters may be contaminated by direct excretion by bathers (vomits, urine, *etc.*), transport on the body or growth within the filter bed [5] and by waterborne pollutants from external sources (*e.g.*, sewage, storm water, and agricultural runoff) [3, 6]. Most microorganisms which are usually connected to swimming pools include *Salmonella typhi, Salmonella paratyphi, Shigella dysenteriae, Vibrio cholera, Pseudomonas aeruginosa, Mycobacterium spp., Staphylococcus aureus,* Legionellae, *Cryptosporidium parvum,* Giardia, Microsporidia, Dermatophytes *and Keratinophilic fungi and Molluscum contagiosum,* hepatitis A and E [2, 7, 8]. This means that unsafe swimming pool water can transmit pathogenic bacteria, fungi, parasites and viruses which can cause ear and eye infections, digestive system infections, skin diseases, infections of the upper respiratory tract in swimmers and systemic infections especially among those who are immune suppressed individuals [1, 6].

If there is no control over health standards for swimming pools, they can be a serious source of microbial contamination since wide range of people with different levels of economic, social and health status use swimming pools [5]. To monitor and control the quality as well as microbial safety related to fecal pollution microbial examination of swimming pools, water is used [9]. To do so since pathogenic microorganisms that can be transmitted by contaminated swimming pools water are diverse and due to the difficulty and cost of directly measuring all microbial pathogens, indicator organisms that may indicate the presence of fecal contamination are often used for monitoring and regulation of recreational waters [9]. As indicators of fecal pollution, *Escherichia coli* presence is a strong indication of the presence of enteric pathogenic bacteria such as *Salmonella typhi, Salmonella paratyphi, Shigella dysenteriae, Vibrio cholerae* and parasites in the pool [2, 8]. These indicator organisms are common inhabitants of the intestinal tract of warm-blooded animals and they are found in feces at high concentration and are easier to measure in the environment than pathogens although they represent a measure of faecal contamination [9]. Hence this study aimed at assessing the physicochemical and microbiological quality of public swimming pools in Addis Ababa and their health significance as there is no such study conducted before in this respect in Ethiopia.

## MATERIALS AND METHOD

A cross sectional study was carried out from February to May, 2016 in Addis Ababa, the capital city of Ethiopia with a population of 3,384,569 according to the 2007 population census with annual growth rate of 3.8%. Addis Ababa culture and tourism bureau gave recognition for nine hotels and one recreation center to have swimming services for the public where only two of these swimming pools were indoors while the rest were outdoors. In this study, all these ten swimming pools which were recognized by Addis Ababa culture and tourism bureau were included.

A total of 60 water samples were collected from ten swimming pools (6 samples from each swimming pool, on a weekly basis). Swimming water samples were collected at three points (from deeper point, shallow point and intake point) from each swimming pool. A 250 ml sterile bottle with sodium thiosulphate was immersed to an elbow depth with its opening facing the water. Its opening was then inverted so that water could get in to it. After the bottle was full, it was withdrawn from the water. PH, residual chlorine and temperature of samples obtained from each point were measured at the time of collection. Sample containing bottles were transported in ice box to microbiological laboratory within two hours of collection. All the samples were analyzed on the day of collection.

## Microbiological Analysis of Swimming Pool Water

**Total Viable Count**: after mixing bottles containing swimming pool water sample by gentle inversion, 1ml of sample was poured in to a sterile culture plate. Then, 20 ml of plate count agar (PCA) was poured on to the plate. The sample and the PCA were mixed and were made to stay at room temperature until the mixture solidified. All plates were incubated for 48 hours at 37 degrees Celsius and total viable counts were then counted by using digital colony counter (24).

**Total Coliform Count:** Three sets of test tubes (one 50ml, five 10ml, five 5 ml) containing Durham’s tubes within them were arranged. Double strength MacConkey broth was poured in to the 50 ml and the 10 ml test tubes (50ml in to the 50 ml and 10 ml in to the 10 ml) while 1 ml of single strength MacConkey broth was added in to each of the 5ml test tubes. Pool water was then added to the three sets of test tubes(50ml in to 50 ml test tube, 10 ml in to each 10 ml,1ml in to each 5 ml test tubes).The contents of each test tube were then mixed. Every test tube was observed for the presence of air bubbles in the Durham’s tubes and air bubbles were removed from test tubes which had them. The lids of each test tube were then loosened and all the tubes were incubated at 37 degrees Celsius. After 48 hours, each test tube was examined for gas production and for change in color. Test tubes which became yellow and produced gas were considered positive for total coliform and those with no color change and gas production were considered negative for total oliform. Finally, the total coliform count was obtained from the Most Probable Number (MPN) .

**Fecal coliform:** Each positive test tube from each set of test tubes was sub-cultured in to test tubes containing *Escherichia coli* broth with Durham`s tube. Contents were mixed and air bubbles were removed from the test tubes. Lids of each test tube were loosened and the test tubes were incubated at 44.4 degrees Celsius for 24 hours, and then all the test tubes were examined for turbidity and gas production. Those with turbidity and gas production were taken as positive for fecal coliforms and the fecal coliform count was determined from the Most Probable Number (MPN).

***Escherichia coli:*** Samples from positive test tubes for fecal coliform were inoculated into nutrient broth and incubated at 44.4 degrees Celsius for 24 hours. After 24 hours incubation, a drop of Kovacs reagent was added and Formation of red ring was used as a proof for indole positivity and hence for the presence of *E. coli* .

### Quality Control

Standard Operating Procedures (SOP) were strictly followed verifying that media meet expiration date and quality control parameters and manufacturer`s instructions regarding media preparation were followed meticulously. Visual inspections of cracks in media or plastic Petri-dishes, unequal fill, evidence of freezing, bubbles, and contamination were carried out. Quality control was performed to check the quality of medium and each new lot was quality controlled before use by testing *Escherichia coli* ATCC 25922 standard control strains.

### Data Analysis

Data were entered, cleaned and analyzed by SPSS version 20. Descriptive statistics (mean, frequency, percent and range) were calculated and a Chi square test was used to check association between the variables. Results were then presented in words and depicted in tables and diagrams.

### Ethical Considerations

An ethical clearance was obtained from departmental research and review committee of School of Medical Laboratory Science, Addis Ababa University. Purpose of the research was then clearly explained by the principal investigator to hotels with swimming pools in Addis Ababa. Persons in charge of the hotels were also informed that information fetched from the swimming pools would be kept confidential. Finally informed consent was obtained from the respective hotels and data collection was started after then.

## RESULTS

### Physicochemical Parameters of the Swimming Pools Water

**Fig. (1):**
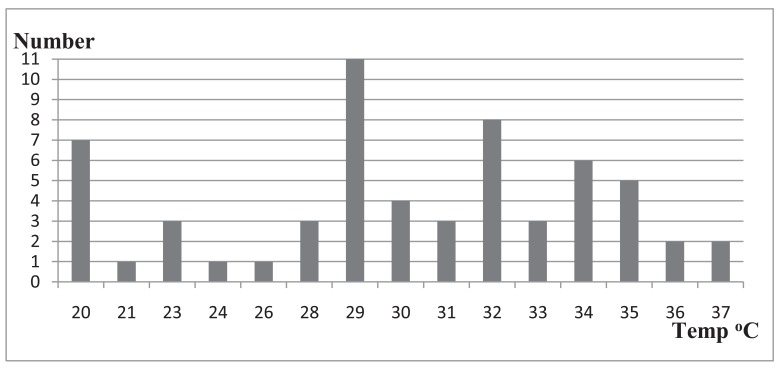
Temperature (^o^C) of swimming pools during study periods.

The average PH value of the swimming pool water samples was 7.1 with a range of 5-10. Among the tested swimming pool water samples, 58.4% (n=35/60) had PH values within WHO recommended limit of 7.2-7.8 while 41.6% (n=25/60) of the water samples had pH values out of the recommended limits where 20% (n=12/60) and 21.7% (n=13/60) were below 7.2 and above 7.8, respectively. The residual chlorine of swimming pools water samples was between 0.0 and 4.0 mg/L where 73.3% (n=44/60) of swimming pool water samples had residual chlorine values in the range of 0.0-1.9mg/L while only 1.7% (n=1/60) of the swimming pool water sample was found to have residual chlorine values of 4mg/L. Majority 73.3% (n=44/60) of the swimming pool water samples had residual chlorine values below 2.0 which is the WHO minimum recommended limit whereas 25% (n=15/60) had values within the WHO recommended range (2-3mg/L) (Table **1**). The study has shown that swimming pool water samples had temperature values ranging from 20^o^C-37^o^C with an average of 29^o^C. More than half *i.e.* 58.3% of (n=35/60) the swimming pool water samples were found to have temperatures ranging from 21^o^C-32^o^C.However, 11.7% (n= 7 /60) of the swimming pool water samples had a temperature of 20^o^C. (Fig. **1**).

### Microbial Assessment of Swimming pool Water Samples

Swimming pool water samples had a total viable count values in the range of 0 cfu/ml-7.6x10^4^ cfu/ml where 73.3% (n=44/60) of swimming pool water samples had a total viable count below 2x10^2^ CFU/ml while 26.7% (n=16/60) of the swimming pool water samples had a total viable count above 2x10^2^ CFU/ml. Regarding total coliform counts (TCC) done on the 60 water samples, 66.7% (n=40/60) of the swimming pool water samples had TCC values of 0 MPN /100ml. Swimming water samples which met the WHO standard with a TCC value of less than 2 MPN /100ml were 70.0% (n=42 /60) while 30% (n=18/60) did not show TCC values above 2 MPN /100ml.

The fecal coliform counts ranged from 0 MPN /100ml -1.8x10^2^ MPN /100ml and 66.7% (n=40/60) swimming pool water samples had fecal coliform counts falling below 1 MPN /100ml while 33.3% (n=20/60) had counts above 1 MPN /100ml. 8.3% (n=5/60) of the swimming pool water samples were found to have a fecal coliform count of 1.8x10^2^ MPN /100ml and 5% (n=3/60) swimming pool water samples had fecal coliform counts of 2.5x10^1^ MPN /100ml, 2x10^1^ MPN /100ml and 1.8x10^1^ MPN /100ml while the rest had counts below the aforementioned values. The swimming pool water samples were also checked for the presence or absence of *E. coli.* Accordingly, *E. coli* was present in 30% (n=18/60) of swimming pool water samples while it was absent in 70% (n=42/60) of the pool water samples.

Overall, the compliance of swimming pool water samples was evaluated against the WHO standard and 68.3% (n=41/60) of the swimming pool water samples which had neither *E. coli* nor fecal coliform showed compliance while 31.7% (n=19/60) of samples which had either *E. coli* or fecal coliform did not comply to the WHO standard. (Table **2**).

## DISCUSSION

Average PH value of 7.1 of swimming pool water samples with a range from 5-10 was comparable to the findings of a study from Iran [10]. Another study from Iran showed a similar range of PH of swimming pools to that of our study finding [11]. Our study showed that 58.4% of the swimming pool water samples complied to the WHO standard for PH (PH:7.2-7.8) while 41.6% had values out of the standard where 20% and 21.6% of them had values below 7.2 and above 7.8, respectively. This may be attributed to the absence of educated pool operators, bathers not taking pre-swim showers and less frequent changing of the pool water.

The residual chlorine of swimming pools water was in the range of 0 to 4mg/l with an average value of 1.16 mg/L which was in accordance with a finding from Iran [10]. This value was in close agreement with another study [12]. Compared to WHO residual chlorine values (2-3mg/l), 73.3% (n=44/60) of the swimming pool water samples showed residual chlorine values in the range of 0-1.9mg/l below the lower WHO recommended limit. Only 25% of the swimming pools water samples had residual chlorine values falling within the WHO recommended range. This suggested that there was no careful monitoring of residual chlorine in the majority of swimming pools.

With regard to temperature, the study indicated that swimming pool water samples had an average temperature of 29^o^C which was in close agreement with the result of another study [10]. Compared with WHO`s recommended temperature range 21^o^C-32^o^C for swimming pool water, 58.3% (n=35/60) of swimming pool water samples were found to have temperatures range falling within the limit. This implies that there was still a problem in monitoring the temperature of swimming pool waters.

In our study, 73.3% swimming pool water samples with a count of 2x10^2^ cfu/ml met the standard while 26.7% with a count above 2x10^2^ cfu/ml did not meet the set standard. The finding of 26.7% of the swimming pool water samples that did not meet the standard was relatively higher as compared with another study [13]. The disparity in result may be attributed to differences in sample size and differences in pool monitoring.

With regard to total coliform count, data from this study showed that a comparable total coliform counts ranged from 0 MPN /100ml to 1.8x10^2^ MPN /100ml with a study conducted in Iran [10]. With regard to the minimum and maximum values of TCC, however, a gap exists. The gap may be because of variation in the study period, sample size and pool water treatment. Another study also showed TCC value of 1.06x10^1^ cfu/100 ml which was not comparable with the result of this study [14]. When evaluated in line with the WHO standard, 70% of the swimming pool water samples had TCC values below 2 MPN /100ml while 30% did not meet the standard with TCC values above 2 MPN /100ml.

Our study indicated that 33.3% of the swimming pool water samples were positive for fecal coliforms which was a much higher finding than a study from Greece which had 3% fecal coliform counts out of 200 samples [15]. Another study showed that 11% of the total water samples were positive for fecal coliforms which was also much lower than our finding [12]. The variation in result could be due to differences in the study period, sample size, and pool treatment. According to WHO recommendation that fecal coliforms to be below 0 MPN /100ml, 66.7% of the water samples in our study met standard with a fecal coliform count of 0 MPN /100ml while 33.3% did not meet the standard with their values being >0 MPN /100 ml. Out of the 33.3%, 8.3% had fecal coliform counts of 1.810^2^ MPN /100ml while 5% had values of 2.5x10^1^ MPN /100ml, 2x10^1^ MPN /100ml and 1.8x10^1^ MPN /100ml.

Regarding the presence or absence of *E. coli,* 30%√(n=18/60) of the samples were positive for *E. coli* while from 70% (n=42/60) water samples, *E. coli* were not detected thus meeting the WHO standard. From a study [11], 16.6% of the samples were positive for *E. coli* which was a lower finding than our study. The non conformance to the standard might be due to differences in the sanitation practice, bather attitude, sample size and study period. According to WHO, for water to be potable, *E. coli*/fecal coliforms should not be detected. In this respect, 68.3% (n=41/60) of the samples had no *E. coli* thus were potable while 31.7% (n=19/60) had *E. coli* and/or *E. coli* indicating non-potability.

## CONCLUSION

Most swimming pools’ water samples showed PH, residual chlorine and temperature value within the acceptable limit recommended by WHO. Good monitoring of residual chlorine, temperature and PH of swimming pools is a must considering their significant impact on the microbial safety of swimming pools which has a health implication on the public who use these pools. Overall, 68.3% (n=41/60) swimming pools water samples complied to the microbial standard which had neither *E. coli* nor fecal coliform. However, swimming pool water samples which had either *E. coli* or fecal coliform that did not comply to the WHO standard indicated the need for swimming pools’ water quality improvement.

## Figures and Tables

**Table 1:** PH and residual chlorine of swimming pools’ water samples.

**Physicochemical characteristics**	**Range**	**Frequency(No.)**	Percent ***(%)***
	5-7.1	12	20
	7.2-7.8	35	58.3
**PH range**	8-10	13	21.7
	**Total**	**60**	**100**
	0-1.9	44	73.3
**Residual**	2-3.9	15	25.0
**Chlorine**	4-4.9	1	1.7
	**Total**	**60**	**100.0**

**Table 2:** Frequency distribution of total viable count, total coliform count, Fecal Coliform and *E. coli.*

	**Frequency**	**Percent**	
**Total coliform count**			
0	40	66.7	
1	2	3.3	
2	2	3.3	
3	1	1.7	
5	1	1.7	
7	1	1.7	
8	3	5.0	
11	2	3.3	
18	1	1.7	
20	1	1.7	
25	1	1.7	
180	5	8.3	
**Total viable count**			
<200	44	73.3	
>200	16	26.7	
**Fecal coliform count**			
0	40		66.7
1	3		5.0
2	2		3.3
3	1		1.7
7	1		1.7
8	3		5.0
11	2		3.3
18	1		1.7
20	1		1.7
25	1		1.7
180	5		8.3
***E. coli* /Fecal coliform**			
Present	19		31.7
Absent	41		68.3
**Total**	**60**		**100.0**
